# Context-Aware Systems for Chronic Disease Patients: Scoping Review

**DOI:** 10.2196/10896

**Published:** 2019-06-17

**Authors:** Kathleen Yin, Liliana Laranjo, Huong Ly Tong, Annie YS Lau, A Baki Kocaballi, Paige Martin, Sanjyot Vagholkar, Enrico Coiera

**Affiliations:** 1 Centre for Health Informatics Australian Institute of Health Innovation Macquarie University Sydney Australia; 2 Macquarie University Health Sciences Centre Macquarie University Sydney Australia

**Keywords:** self-care, medical informatics, mobile applications, chronic disease, self-management

## Abstract

**Background:**

Context-aware systems, also known as context-sensitive systems, are computing applications designed to capture, interpret, and use contextual information and provide adaptive services according to the current context of use. Context-aware systems have the potential to support patients with chronic conditions; however, little is known about how such systems have been utilized to facilitate patient work.

**Objective:**

This study aimed to characterize the different tasks and contexts in which context-aware systems for patient work were used as well as to assess any existing evidence about the impact of such systems on health-related process or outcome measures.

**Methods:**

A total of 6 databases (MEDLINE, EMBASE, CINAHL, ACM Digital, Web of Science, and Scopus) were scanned using a predefined search strategy. Studies were included in the review if they focused on patients with chronic conditions, involved the use of a context-aware system to support patients’ health-related activities, and reported the evaluation of the systems by the users. Studies were screened by independent reviewers, and a narrative synthesis of included studies was conducted.

**Results:**

The database search retrieved 1478 citations; 6 papers were included, all published from 2009 onwards. The majority of the papers were quasi-experimental and involved pilot and usability testing with a small number of users; there were no randomized controlled trials (RCTs) to evaluate the efficacy of a context-aware system. In the included studies, context was captured using sensors or self-reports, sometimes involving both. Most studies used a combination of sensor technology and mobile apps to deliver personalized feedback. A total of 3 studies examined the impact of interventions on health-related measures, showing positive results.

**Conclusions:**

The use of context-aware systems to support patient work is an emerging area of research. RCTs are needed to evaluate the effectiveness of context-aware systems in improving patient work, self-management practices, and health outcomes in chronic disease patients.

## Introduction

### Background

As health care moves from the traditional hospital setting to the personal sphere of home and community, individuals are increasingly being encouraged to engage in self-care [1,2]. Sociologists refer to this act of self-care as *patient work*, which involves effort and investment of time on the part of patients or family members to accomplish a health goal [3]. Patient work extends beyond strictly health-related tasks and is shaped by the context of patients’ lives and their daily routines [1]. It has been suggested that the use of context-aware technologies may thus better support patient work and improve self-care, as contextual information could trigger more personalized and relevant services or information [4].

Context-aware systems, also known as context-sensitive systems, are computing applications designed to capture, interpret, and use contextual information and provide adaptive services according to the current context of use [4,5]. Context-aware systems may thus harness everything from sensors that capture data indicative of context (such as time, location, and light intensity) to inference mechanisms that interpret and action such data [5]. Even though context-aware systems have been piloted in some health care settings, their impact on health care outcomes remains unclear. Specifically, context-aware systems have mainly been piloted in the hospital setting [4] and for primary prevention [6-8], rarely addressing the context of chronic disease patients’ health-related activities in everyday life.

### Objectives

The aim of this study was to examine existing literature on interventions using context-aware technologies that support *patient work*. Specifically, we sought to characterize the different tasks and contexts in which such systems were used, as well as assess any existing evidence about their impact on health-related process and outcome measures.

## Methods

### Search Strategy

A systematic search of the literature was performed in September 2016 and updated in October 2017 on MEDLINE, EMBASE, CINAHL, ACM Digital, Web of Science, and Scopus using search terms regarding patient work, context awareness, and consumer health informatics. The complete search strategy is available in Multimedia Appendix 1. The reference lists of relevant articles were also screened to ensure that all eligible studies were captured. A grey literature search was performed using Google Scholar to capture dissertations, theses, and conference proceedings that met the inclusion criteria.

### Study Selection Criteria

In the scope of our study, we focused on context-aware systems that were capable of (1) capturing and processing contextual information (eg, environmental data and user-related features) and (2) using the captured contextual information to provide adaptive services and support patient work tasks in everyday life, either at home or in the community.

Studies were included in the review if they focused on patients with chronic conditions, involved the use of a context-aware system to support patients’ health-related activities, and reported the evaluation of the systems by the users.

Studies were excluded if they were not in English or if they focused on health care providers instead of consumers. We also excluded interventions that merely gathered and displayed context information, without using it to adapt system behavior (passive context awareness), as this was outside the scope of this study.

### Paper Screening Process

We conducted a 2-phase screening process, initially excluding papers based on their titles and abstracts using a standard screening form, and then rescreening the remaining papers based on the full-text article.

Both phases were conducted by teams of 2 independent reviewers (2 teams in the first phase and 1 in the second). Cohen kappa was used in the full-text paper screening to measure intercoder agreement. Any disagreements in the screening were resolved through discussion and consensus.

### Data Extraction and Synthesis Strategy

One reviewer extracted information from the eligible studies into a data extraction form, whereas 2 other reviewers examined the completed form for consistency and accuracy. The following information was collected: first author, year, health domain, study type, participants’ characteristics (number, age, and sex ratio), intervention characteristics, health activities (tasks undertaken by patients to achieve health goals), and main findings. To explore how context was utilized by the included studies, we analyzed the related contextual elements based on previous work [4,9]. Context information was grouped into the following dimensions: setting (indoor or outdoor), environmental features (indoor and outdoor attributes, eg, room temperature, humidity, and air pollen), and user features (user-related data captured by the system, eg, physical activity, physiological measurements, and mental state). Time is also considered an important element, but it is often coupled with other dimensions, so it was not analyzed separately in this review. Finally, we characterized the utilization of context for each study (ie, the adaptive services provided by the system based on contextual data).

Our study design follows the guidelines for a scoping review proposed by Arksey and O’Malley (2005) and follows the Preferred Reporting Items for Systematic Reviews and Meta-Analyses statement [10], where applicable.

## Results

### Retrieved Studies

**Figure 1:**
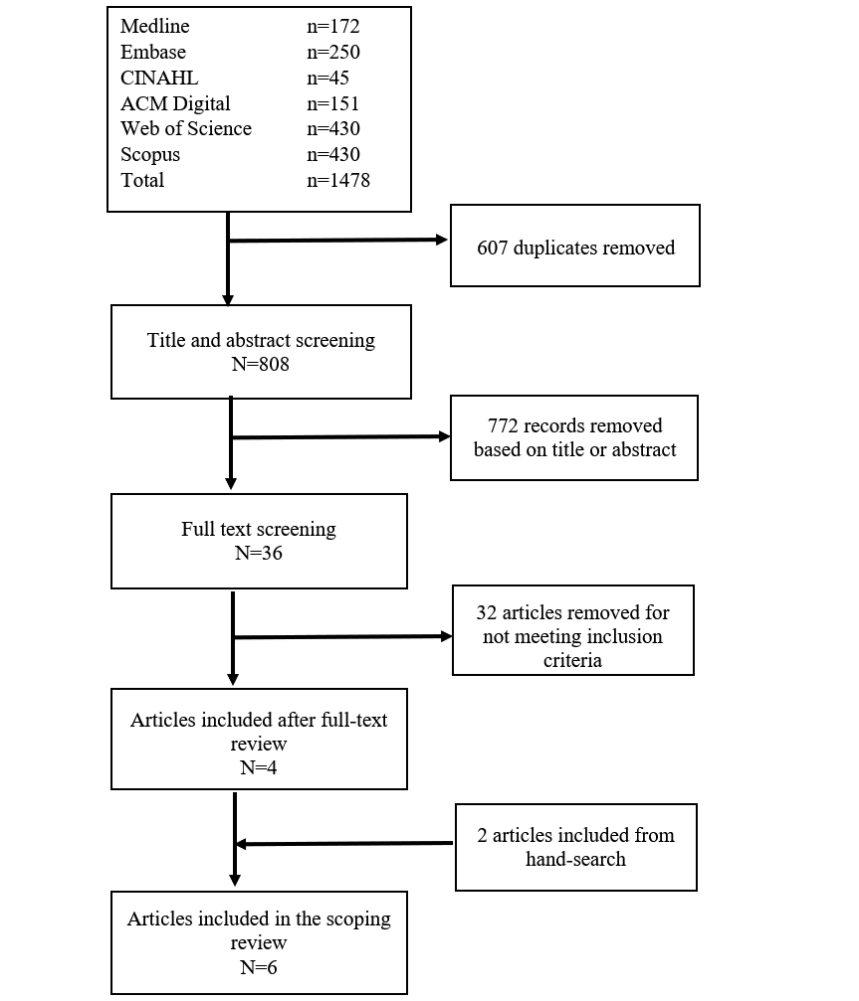
Flow diagram of included studies.

The database search retrieved 1478 citations; 607 duplicates were removed (Figure 1). After screening the abstracts and titles, 768 articles were excluded for not meeting the eligibility criteria. Full-text screening eliminated 36 articles (a list of excluded articles is available in Multimedia Appendix 2). An additional 2 articles were found via hand-search, yielding 6 included studies in total. The kappa statistic measuring inter-rater agreement for full-text screening was 0.6 (moderate agreement) [11].

### Description of Included Studies

The number of participants in studies ranged from 4 to 47 (Table 1). A total of 1 study was conducted in Canada [12], 2 in the United States [13,14], and 2 in Europe [15,16]. Most articles were published after 2010 except 1 [17]. The health domains covered in the studies included asthma [13], cardiovascular disease [16], kidney disease [12], Parkinson disease [17], diabetes [15], and mental health [14]. All included studies used quasi-experimental study designs to pilot test different context-aware systems. Demographic information on participants was often missing and inconsistently reported. Specifically, age data were reported by 3 studies [12,14,17], and 4 studies reported sex data [12,14,15,17].

**Table 1:** Characteristics of included studies and context-aware systems.

Study author, year, location	Health domain	Study type; duration	N total (mean age, % female)	Health activities	Patient-facing technologies	Functions
Bächlin et al, 2009, Israel [17]	Parkinson disease	Quasi-experimental; not reported (NR)	10 patients (66.4, 30)	Self-management of gait deficits in Parkinson patients	Acceleration sensors	Detect movement and freezing of gait
					Earphones and wearable computer	Produce sound when freezing of gait occurs (continuous external rhythmic auditory cues improve gait performance)>
Ong et al, 2016, Canada [12]	Chronic kidney disease	Quasi-experimental; 6 months	47 patients with chronic kidney disease (59, 47)	Self-management of chronic kidney disease (self-monitor blood pressure [BP] and symptoms, manage medications, track lab test results)	Wireless BP monitor	Measure BP
					Mobile app	Personalized real-time feedback on BP; reminders (eg, reconcile medication, and measure BP); self-monitor symptoms; access to lab test results and medication list
Lamprinos et al, 2016, Germany and Turkey [15]	Diabetes	Quasi-experimental; 6 weeks	In Germany: 21 patients (NR, 24); In Turkey: 39 patients (NR, 46)	Self-management of diabetes (self-monitor physiological measures; manage medications and lifestyle behaviors)	Mobile app and website	Self-monitor (eg, blood glucose, weight, BP, medication, physical activity, diet, and sleep); personalized feedback (decision making and action planning)
Zhang et al, 2016, Germany [16]	Cardiovascular disease	Quasi-experimental; NR	5 healthy young adults (NR)	Self-management of cardiovascular disease (self-monitor heart rate and identify abnormalities)	Wearable sensors	Track physical activity, heart rate, skin temperature, cardiac and pulmonary function, posture
					Environmental sensors	Detect room temperature
					Mobile app	Retrieve sensor data; trigger an alarm when an abnormal heartbeat is detected
Anantharam et al, 2015, United States [13]	Asthma	Quasi-experimental; 10 days	4 children (NR)	Self-management of asthma (self-monitor symptoms and identify triggers)	Indoor sensor	Monitor environmental and air quality observations (eg, pollen levels, carbon monoxide, temperature, and humidity)
					Exhaled air sensor	Monitor exhaled nitric oxide (indicator of inflammation)
					Mobile app	Gather and display sensor data; record users’ observations (eg, asthma-related symptoms) via questionnaires; personalized feedback
Burns et al, 2011, United States [14]	Major depressive disorder	Quasi-experimental; 8 weeks	8 patients (37.4, 88)	Self-management of depression (self-monitor symptoms and identify triggers)	Mobile phone sensors	Collect data on location, ambient light, phone usage
					Website	Provide behavioral therapy; display data collected from the mobile phone
					Mobile app	Collect self-reported data on social context, activity, location, and internal states (ie, mood) via ecological momentary assessment; integrate self-reports with sensor data; personalized feedback; predict patient states based on self-reports and sensor data

### Context Elements and Technologies of Current Interventions

The contextual elements of each included study are summarized in Table 2. A total of 2 studies focused on indoor settings [13,17], and 4 studies involved both indoor and outdoor settings [12,14-16]. Context was captured using sensors [12-14,16,17] and self-reports [12-15]. A total of 1 particular study used ecological momentary assessment to capture self-reported data on social context, activity, and internal states (ie, mood) [14]. Sensors captured data on location, ambient light [14], air quality [13], room temperature [16], and physiological measures (blood pressure [BP], heart rate, skin temperature, cardiac function, exhaled nitric oxide) [12,13,16]. Acceleration sensors were used to track movement and physical activity [16,17].

**Table 2:** Context elements present in included studies.

Study, year	Settings	Environmental features	User features	Utilization of context
Bächlin, 2009 [14]	Indoor	None	Movement tracking	Real-time movement tracking system triggering cueing sound upon detection of freezing of gait.
Ong, 2016 [12]	Indoor and outdoor	None	Blood pressure (BP)	Provide real-time personalized feedback on BP (eg, uncontrolled BP triggered reminder messages recommending an increase in frequency of self-monitoring).
Lamprinos, 2016 [15]	Indoor and outdoor	None	Physical activity tracking (step counts), sleep tracking, blood glucose, BP, weight, mood, nutrition	Creates a personalized action plan based on patient-recorded data and generates self-management recommendations.
Zhang, 2016 [16]	Indoor and outdoor	Room temperature	Physical activity tracking (standing, walking, running, jumping, walking upstairs or downstairs), heart rate, skin temperature, cardiac function, pulmonary function, posture	Trigger an alarm when an abnormal heartbeat is detected.
Anantharam, 2015 [13]	Indoor	Carbon monoxide, temperature, humidity, pollen levels	Exhaled nitric oxide, asthma-related symptoms (eg, coughing and chest tightness**)**	Provide personalized actionable recommendations based on sensor data and patient-reported information (eg, identify and alert patients regarding triggers).
Burns, 2011 [13]	Indoor and outdoor	Location sensing, ambient light	Physical activity tracking, social context (eg, interactions with other people), and internal states (mood, intensity of discrete emotions, fatigue, sense of accomplishment, concentration and engagement, and perceived control over current activities); manually self-reported via ecological momentary assessment	Predict patient states based on self-reported and sensor data (using machine learning), displaying them on the mobile app. Future iterations will involve the use of predicted states to provide real-time interventions.

Most studies used a combination of sensor technology and mobile apps, where the sensors collected context information, and the apps utilized those data to deliver personalized feedback [12-14,16]. Only 1 study did not involve the use of sensors, collecting contextual information solely through user self-reports [15]. In another intervention, a sensor was used without a mobile app, where context was harnessed with the help of a wearable computer, and earphones delivered auditory cues to improve gait performance in Parkinson patients [17].

### Health Activities and Health-Related Measures

Self-monitoring was the most frequent health activity supported by context-aware systems in the included studies, where the collected data were then used to provide personalized feedback. Self-monitored data included physiological measures (eg, BP) [12,13,15,16], symptoms [12-14], and lifestyle behaviors [15]. Other health activities included tracking lab test results [12], managing medications [12,15], and practicing specific behaviors (eg, overcoming freezing of gait) [17].

Only 3 studies reported the impact of the intervention on health-related measures [12,14]. Specifically, Bächlin [17] found that the intervention had a sensitivity of 73.1% and a specificity of 81.6% in detecting freezing of gait events. Ong et al [12] found statistically significant reductions in home BP readings between baseline and after intervention (systolic BP: –3.4 mmHg; 95% CI –5.0 to –1.8; diastolic BP: –2.1 mmHg; 95% CI –2.9 to –1.2). Burns et al [14] found a significant decrease in self-reported depressive symptoms (*P*<.001; per-­protocol Cohen *d*=3.43) and comorbid anxiety symptoms (*P*<.001, per-­protocol Cohen *d*=2.58) [14]).

No studies mentioned a thorough evaluation of patient safety problems. A total of 4 studies highlighted technical issues such as system downtime [15], battery drainage problems [13,14], and wearable sensor issues in activity detection [16].

## Discussion

### Principal Findings

To the best of our knowledge, this is the first systematic scoping review to examine context-aware interventions to support patient work. The emerging nature of the field is reflected in the small number of included studies, their recent time of publication (all after 2010), and the predominance of quasi-experimental study designs. The majority of the papers involved pilot and usability testing with a small number of users; there were no randomized controlled trials (RCTs) to evaluate the efficacy of a context-aware system. In the included studies, context was captured using sensors or self-reports, sometimes involving both. Most studies used a combination of sensor technology and mobile apps to deliver personalized feedback. A total of 3 studies examined the impact of interventions on health-related measures, showing moderate-to-good sensitivity and specificity in detecting freezing of gait events in Parkinson patients [17], as well as significant improvements in BP [12] and reductions in depression symptoms and comorbid anxiety symptoms [14].

### Comparison With Previous Literature

Other reviews have looked at the use of context awareness in health care [4,18]. Bricon-Souf (2007) found that there was a large gap between the requirements expressed by users and the context-aware prototypes developed. In addition, they reported that there was no consensus in the research community on how to model context and architectures to support its use. Similarly, Orwat et al revealed that most systems were described in their prototype stage and that implementation issues were rarely mentioned.

Our study, though only focused on patient work, also revealed comparable findings. Most studies described prototypes, and only 3 studies examined the impact of interventions on health-related measures, showing promising results in detecting freezing of gait events in Parkinson patients [17], as well as in improving BP [12] and depression and anxiety symptoms [14]. The use of context-awareness systems in patient work interventions has the potential to facilitate self-monitoring and improve the relevance and quality of the feedback provided, personalizing it to better fit participants’ context [12-14,16]. This sort of “just-in-time” support [19] has the potential to facilitate patient work and improve the self-management of chronic conditions, by providing the advice patients need to make health management decisions at the right time, on a daily basis. Ameliorating self-management practices is a cornerstone of quality improvement efforts in chronic disease care and is associated with better health outcomes in several conditions such as type 2 diabetes [20].

The costs and risks of using context-aware systems for patient work were rarely reported in the included studies. A total of 4 studies highlighted technical issues such as system downtime [15], battery drainage problems [13,14], and wearable sensor issues in activity detection [16]. No studies mentioned a thorough evaluation of patient safety problems. Future studies should consistently report unintended effects and possible harms of the systems, such as privacy, technical issues, or any other unanticipated incidents [21].

### Strengths and Limitations

This systematic scoping review has several strengths in terms of study design. First, an extensive search was performed across multiple databases to ensure that all relevant studies were captured. Second, the screening form was pretested and piloted before screening. Third, all full-text papers were screened by 2 independent reviewers. Finally, the kappa score of 0.6 for the full-text screening phase revealed an acceptable level of agreement.

The results of our study need to be interpreted in light of some limitations. Given that this is an emerging field in health informatics, there is a lack of longitudinal and experimental studies, which hampers the evaluation of the impact of these interventions. This is the reason why a systematic scoping review was conducted instead of a systematic review.

Another limitation was the exclusion of non-English papers. Even though this was conducted to ensure that all the authors could adequately understand and make an informed decision based on the abstracts, we might have missed important papers on patient work.

### Implications for Research and Health Care

The use of context-aware systems to support patient work is a promising area of research, as these interventions have the potential to facilitate self-monitoring and provide personalized just-in-time feedback based on users’ characteristics and environmental features, with the aim of improving disease management and clinical outcomes. Specifically, the increasing use of sensors to automatically collect context information could eliminate the need for self-reporting and manual data entry, streamlining the task of self-monitoring for chronic disease patients [22]. Furthermore, future applications of artificial intelligence have the potential to expand on the current capacity of these systems to provide personalized and relevant services to individuals [23], better supporting users with their health-related tasks, and decreasing the burden of patient work.

A common issue in context-aware systems research is the challenge of evaluating their real-world implementation [4,18]. Implementation fidelity is “the degree to which programs are implemented as intended by the program developers” [24]. It is known that implementation settings play a crucial role in the effectiveness of interventions, an issue that is at the core of implementation science’s efforts to model the impact of context on outcomes [25]. To allow for implementation fidelity and replicability, studies of context-aware systems should describe the setting explicitly as well as provide sufficient details about the intervention and any potential adaptations for it to fit a different setting [26,27].

### Conclusions

The use of context-aware systems to support patient work is an emerging area of research. RCTs are needed to evaluate the effectiveness of context-aware systems in improving patient work, self-management practices, and health outcomes in chronic disease patients. Future studies should consistently report the intervention and the settings in which the intervention is being implemented.

## Appendix

Multimedia Appendix 1 Search strategy.

Multimedia Appendix 2 Excluded articles.

## Abbreviations

BP: blood pressure
RCT: randomized controlled trial
