# Supplementary material for: Context-Aware Systems for Chronic Disease Patients: Scoping Review
Source: J Med Internet Res. 2019 Jun 17;21(6):e10896. doi: 10.2196/10896 (PMC6601254; doi:10.2196/10896)
Supplement: Multimedia Appendix 1 [file jmir_v21i6e10896_app1.pdf]

Supplementary Information File 1

1.1 Search strategy for MEDLINE (via PubMed interface)

URL: https://www.ncbi.nlm.nih.gov/pubmed/
Limits: none
Conducted in September 2016 and updated in October 2017
#1 ("Medical Informatics"[Mesh] OR "Consumer Health Information"[Mesh] OR "digital health" or "ehealth" OR "information technology" OR "information communication technology" OR "decision support system" OR "Health informatics" OR "medical informatics" OR "decision support") **AND**

#2 ("consumer work" OR "consumer activit*" OR "consumer task*" OR "consumer schedule*" OR "consumer context*" OR "consumer routine*" OR "consumer self-management" OR "consumer self-monitor*" OR "consumer self-care") OR (“patient work” OR “patient activit*” OR “patient task*” OR “patient schedule*” OR “patient context*” OR “patient routine*” OR “patient self-management” OR “patient self-monitor*” OR “patient self-care”)OR (“carer work” OR “carer activit*” OR “carer task*” OR “carer schedule*” OR “carer context*” OR “carer routine*”) OR ("Caregivers" [Mesh] AND (work OR activit* OR task* OR schedule* OR routine*)

1.2 Search strategy for Embase

URL: Macquarie University Library (via OVID Interface)
Limits: none
Conducted in September 2016 and updated in October 2017
#1 ("Medical Informatics"[Mesh] OR "Consumer Health Information"[Mesh] OR "digital health" or "ehealth" OR "information technology" OR "information communication technology" OR "decision support system" OR "Health informatics" OR "medical informatics" OR "decision support") **AND**
#2 ("consumer work" OR "consumer activit*" OR "consumer task*" OR "consumer schedule*" OR "consumer context*" OR "consumer routine*" OR "consumer self-management" OR "consumer self-monitor*" OR "consumer self-care") OR (“patient work” OR “patient activit*” OR “patient task*” OR “patient schedule*” OR “patient context*” OR “patient routine*” OR “patient self-management” OR “patient self-monitor*” OR “patient self-care”) OR (“carer work” OR “carer activit*” OR “carer task*” OR “carer schedule*” OR “carer context*” OR “carer routine*”) OR ("Caregiver" [Mesh] AND (work OR activit* OR task* OR schedule* OR routine*)

1.3 Search strategy for CINAHL

URL: Macquarie University Library (via OVID Interface)
Limits: none
Conducted in September 2016 and updated in October 2017
#1 ("Medical Informatics" OR "Consumer Health Information" OR "digital health" or "ehealth" OR "information technology" OR "information communication technology" OR "decision support system" OR "Health informatics" OR "decision support") **AND**
#2 ("consumer work" OR "consumer activit*" OR "consumer task*" OR "consumer schedule*" OR "consumer context*" OR "consumer routine*" OR "consumer self-management" OR "consumer self-monitor*" OR "consumer self-care" OR“patient work” OR “patient activit*” OR

“patient task*” OR “patient schedule*” OR “patient context*” OR “patient routine*” OR “patient self-management” OR “patient self-monitor*” OR “patient self-care” OR “carer work” OR “carer activit*” OR “carer task*” OR “carer schedule*” OR “carer context*” OR “carer routine*”OR "caregiver work” OR “caregiver activit*” OR “caregiver task*” OR “caregiver schedule* OR “caregiver routine*”)

1.4 Search strategy for ACM Digital
URL: Macquarie University Library (via OVID Interface)
Limits: none
Conducted in September 2016 and updated in October 2017
#1 ("Medical Informatics" OR "Consumer Health Information" OR "digital health" or "ehealth" OR "information technology" OR "information communication technology" OR decision support system" OR "Health informatics" OR "decision support") **AND**
#2 ("consumer work" OR "consumer activit*" OR "consumer task*" OR "consumer schedule*" OR "consumer context*" OR "consumer routine*" OR "consumer self-management" OR "consumer self-monitor*" OR "consumer self-care") OR (“patient work” OR “patient activit*” OR “patient task*” OR “patient schedule*” OR “patient context*” OR “patient routine*” OR “patient self-management” OR “patient self-monitor*” OR “patient self-care”) OR (“carer work” OR “carer activit*” OR “carer task*” OR “carer schedule*” OR “carer context*” OR “carer routine*”) OR ("caregiver work” OR “caregiver activit*” OR “caregiver task*” OR “caregiver schedule*” OR “caregiver routine*”)

1.5 Search strategy for Web of Science
URL: Macquarie University Library (via OVID Interface)
Limits: none
Conducted in September 2016 and updated in October 2017
#1 ("Medical Informatics" OR "Consumer Health Information" OR "digital health" or "ehealth" OR "information technology" OR "information communication technology" OR "decision support system" OR "Health informatics" OR "decision support") **AND**
#2 ("consumer work" OR "consumer activit*" OR "consumer task*" OR "consumer schedule*" OR "consumer context*" OR "consumer routine*" OR "consumer self-management" OR "consumer self-monitor*" OR "consumer self-care" OR "patient work" OR "patient activit*" OR "patient task*" OR "patient schedule*" OR "patient context*" OR "patient routine*" OR "patient self-management" OR "patient self-monitor*" OR "patient self-care" OR "carer work" OR "carer activit*" OR "carer task*" OR "carer schedule*" OR "carer context*" OR "carer routine*" OR "caregiver work" OR "caregiver activit*" OR "caregiver task*" OR "caregiver schedule*" OR "caregiver routine*")

1.6 Search strategy for Scopus
URL: Macquarie University Library (via OVID Interface)
Limits: none
Conducted in September 2016 and updated in October 2017
#1 ("Medical Informatics" OR "Consumer Health Information" OR "digital health" or "ehealth" OR "information technology" OR "information communication technology" OR "decision support system" OR "Health informatics" OR "decision support") **AND**
#2 ("consumer work" OR "consumer activit*" OR "consumer task*" OR "consumer schedule*" OR "consumer context*" OR "consumer routine*" OR "consumer self-management" OR

"consumer self-monitor*" OR "consumer self-care" OR "patient work" OR "patient activit*" OR "patient task*" OR "patient schedule*" OR "patient context*" OR "patient routine*" OR "patient self-management" OR "patient self-monitor*" OR "patient self-care" OR "carer work" OR "carer activit*" OR "carer task*" OR "carer schedule*" OR "carer context*" OR "carer routine*" OR "caregiver work" OR "caregiver activit*" OR "caregiver task*" OR "caregiver schedule*" OR "caregiver routine*")
