# Supplementary material for: Context-Aware Systems for Chronic Disease Patients: Scoping Review
Source: J Med Internet Res. 2019 Jun 17;21(6):e10896. doi: 10.2196/10896 (PMC6601254; doi:10.2196/10896)
Supplement: Multimedia Appendix 2 [file jmir_v21i6e10896_app2.pdf]

Supplement 2: Excluded studies after full-text revision, for not meeting inclusion criteria

1. Abidi S, Vallis M, Abidi SSR, Piccinini-Vallis H, Imran SA. D-WISE: diabetes web-centric information and support environment: conceptual specification and proposed evaluation. Can J Diabetes 2014;38(3):205-211. PMID: 24909091.
2. Ammerlaan JW, van Os-Medendorp H, de Boer-Nijhof NC, Prakken B, Bijlsma JW, Kruize AA. The most important needs and preferences of patients for support from health care professionals: A reflective practice on (transitional) care for young adults with Juvenile Idiopathic Arthritis. Patient Educ Couns 2017;100(10):1961-1964. PMID: 28363359.
3. Anderson K, Burford O, Emmerton L. Mobile health apps to facilitate self-care: a qualitative study of user experiences. PLoS One 2016;11(5):e0156164. doi: https://doi.org/10.1371/journal.pone.0156164.
4. Boele FW, van Uden-Kraan CF, Hilverda K, Weimer J, Donovan HS, Drappatz J, et al. Neuro-oncology family caregivers' view on keeping track of care issues using eHealth systems: it'sa question of time. J Neurooncol 2017;134(1):157-167. PMID: 28550505.
5. Donsa K, Spat S, Beck P, Pieber TR, Holzinger A. Towards personalization of diabetes therapy using computerized decision support and machine learning: some open problems and challenges. Smart Health: Springer; 2015. p. 237-260.
6. Ferguson G, Quinn J, Horwitz C, Swift M, Allen J, Galescu L. Towards a personal health management assistant. J Biomed Inform 2010;43(5):S13-S16. doi: https://doi.org/10.1016/j.jbi.2010.05.014.
7. Fuentes C, Rodríguez I, Herskovic V, editors. Making communication frequency tangible: How green is my tree? Proceedings of the TEI'16: Tenth International Conference on Tangible, Embedded, and Embodied Interaction; 2016: ACM.
8. García-Sáez G, Rigla M, Martínez-Sarriegui I, Shalom E, Peleg M, Broens T, et al. Patient-oriented computerized clinical guidelines for mobile decision support in gestational diabetes. J Diabetes Sci Technol 2014;8(2):238-246. PMID: 24876573.
9. Georga E, Protopappas V, Guillen A, Fico G, Ardigo D, Arredondo MT, et al., editors. Data mining for blood glucose prediction and knowledge discovery in diabetic patients: The METABO diabetes modeling and management system. Engineering in Medicine and Biology Society, 2009 EMBC 2009 Annual International Conference of the IEEE; 2009: IEEE.
10. Gray CS, Miller D, Kuluski K, Cott C. Tying eHealth tools to patient needs: exploring the use of ehealth for community-dwelling patients with complex chronic disease and disability. JMIR Res Protoc 2014;3(4). PMID: 25428028.
11. Holden RJ, Mickelson RS, editors. Performance barriers among elderly chronic heart failure patients: An application of patient-engaged human factors and ergonomics. Proceedings of the Human Factors and Ergonomics Society Annual Meeting; 2013: SAGE Publications Sage CA: Los Angeles, CA.
12. Milavec MK, Šusteršič O, Rajkovič V. The integrated patient's self-care process model. Stud Health Technol Inform 2016;225:108-112. PMID: 27332172.
13. Karlsudd P. E-collaboration for children with functional disabilities. Telemed J E Health 2008;14(7):687-694. PMID: 18817498.
14. Kim H-S, Jeong YJ, Baik SJ, Yang SJ, Kim TM, Kim H, et al. Social networking services-based communicative care for patients with diabetes mellitus in Korea. Appl Clin Inform 2016;7(03):899-911. PMID: 27679839.

15. Klapper B, Kühne H. Patient self-management by telehealth using the Bosch model of care. J Telemed Telecare 2010;16(4):193-195. PMID: 20511572.
16. Lasorsa I, D'Antrassi P, Ajčević M, Stellato K, Di Lenarda A, Marceglia S, et al. Personalized support for chronic conditions. Appl Clin Inform 2016;7(03):633-645. doi: 10.4338/ACI-2016-01-RA-0011.
17. Lloyd B, Groat D, Cook CB, Kaufman D, Grando A. iDECIDE: A mobile application for insulin dosing using an evidence based equation to account for patient preferences. Stud Health Technol Inform 2015;216:93. PMID: 26262017.
18. Maher M, Kaziunas E, Ackerman M, Derry H, Forringer R, Miller K, et al. User-centered design groups to engage patients and caregivers with a personalized health information technology tool. Biol Blood Marrow Transplant 2016;22(2):349-358. PMID: 26343948.
19. Mao AY, Mathews R, Chen C, Eichstaedt JC, Pendergraft TB, Manassei H, et al. Abstract 18295: Feasibility of a smartphone-based health coaching intervention for patient self-management of risk prevention after acute myocardial infarction. Circulation 2016;134(Suppl 1):A18295-A18295.
20. Martinez VI, Marquard JL, Saver B, Garber L, Preusse P. Consumer health informatics interventions must support user workflows, be easy-to-use, and improve cognition: applying the SEIPS 2.0 model to evaluate patients' and clinicians' experiences with the CONDUIT-HID intervention. Int J Hum Comput Interact 2017;33(4):333-343. doi: https://doi.org/10.1080/10447318.2016.1278340.
21. Masi CM, Suarez-Balcazar Y, Cassey MZ, Kinney L, Piotrowski ZH. Internet access and empowerment. J Gen Intern Med 2003;18(7):525-530. doi: https://doi.org/10.1046/j.1525-1497.2003.20344.x.
22. Menefee HK, Thompson MJ, Guterbock TM, Williams IC, Valdez RS. Mechanisms of communicating health information through facebook: implications for consumer health information technology design. J Med Internet Res 2016;18(8). PMID: 27515151.
23. Or CK, Valdez RS, Casper GR, Carayon P, Burke LJ, Brennan PF, et al. Human factors and ergonomics in home care: Current concerns and future considerations for health information technology. Work 2009;33(2):201-209. PMID: 19713630.
24. Pollack AH, Backonja U, Miller AD, Mishra SR, Khelifi M, Kendall L, et al., editors. Closing the gap: Supporting patients' transition to self-management after hospitalization. Proceedings of the 2016 CHI Conference on Human Factors in Computing Systems; 2016: ACM.
25. Polniaszek S. Long-term care counselor: an electronic decision-support tool. Care Manag J 2004;5(3):139. PMID: 16149251.
26. Qi J, Chen L, Leister W, Yang S, editors. Towards knowledge driven decision support for personalized home-based self-management of chronic diseases. Ubiquitous Intelligence and Computing and 2015 IEEE 12th Intl Conf on Autonomic and Trusted Computing and 2015 IEEE 15th Intl Conf on Scalable Computing and Communications and Its Associated Workshops (UIC-ATC-ScalCom), 2015 IEEE 12th Intl Conf on; 2015: IEEE.
27. Reeder B, Cook PF, Meek PM, Ozkaynak M, editors. Smart Watch Potential to Support Augmented Cognition for Health-Related Decision Making. International Conference on Augmented Cognition; 2017: Springer.
28. Rief JJ, Hamm ME, Zickmund SL, Nikolajski C, Lesky D, Hess R, et al. Using health information technology to foster engagement: Patients' experiences with an active patient health record. Health Commun 2017;32(3):310-319. PMID: 27223684.

29. Rodgers MM, Cohen ZA, Joseph L, Rossi W. Workshop on personal motion technologies for healthy independent living: executive summary. Arch Phys Med Rehabil 2012;93(6):935-939. PMID: 22465525.
30. Valdez RS, Gibbons MC, Siegel ER, Kukafka R, Brennan PF. Designing consumer health IT to enhance usability among different racial and ethnic groups within the United States. Health Technol 2012;2(4):225-233. doi: https://doi.org/10.1007/s12553-012-0031-6.
31. Valdez RS, Holden RJ, Hundt AS, Marquard JL, Montague E, Nathan-Roberts D, et al., editors. The work and work systems of patients: A new frontier for macroergonomics in health care. Proceedings of the Human Factors and Ergonomics Society Annual Meeting; 2014: SAGE Publications Sage CA: Los Angeles, CA.
32. Vargas-Lombardo M, Jipsion A, Vejarano R, Camargo I, Álvarez H, Mora EV, et al., editors. Implementation a Holistic Model, Interactive and Persuasive to facilitate self-care of patients with diabetes (hIPAPD). eHealth, Telemedicine, and Social Medicine, 2010 ETELEMED'10 Second International Conference on; 2010: IEEE.
